# Supplementary material for: Is there any difference between the owners and the public in their visual impact assessments?——A case study of the front garden of multi-storey residential buildings
Source: PLoS One. 2024 Jan 2;19(1):e0296519. doi: 10.1371/journal.pone.0296519 (PMC10760914; doi:10.1371/journal.pone.0296519)
Supplement: S1 File — (DOCX) [file pone.0296519.s001.docx]

Effective questionnaire data:

| **A** | **B** | **C** | **D** | **E** | **F** | **G** | **H** | **I** |
| --- | --- | --- | --- | --- | --- | --- | --- | --- |
| 4 | 5 | 4 | 4 | 1 | 5 | 4 | 5 | 4 |
| 0 | 4 | 5 | 5 | 5 | 4 | 5 | 4 | 5 |
| 4 | 4 | 3 | 5 | 3 | 3 | 4 | 3 | 4 |
| 3 | 5 | 2 | 1 | 1 | 4 | 2 | 5 | 5 |
| 4 | 5 | 5 | 2 | 3 | 3 | 4 | 0 | 2 |
| 0 | 4 | 5 | 3 | 0 | 3 | 4 | 4 | 1 |
| 2 | 5 | 5 | 5 | 5 | 5 | 5 | 5 | 5 |
| 0 | 5 | 0 | 4 | 5 | 3 | 5 | 4 | 4 |
| 3 | 2 | 3 | 5 | 5 | 5 | 5 | 1 | 4 |
| 4 | 5 | 1 | 2 | 5 | 5 | 3 | 4 | 4 |
| 5 | 3 | 3 | 5 | 4 | 4 | 2 | 5 | 5 |
| 4 | 2 | 2 | 0 | 1 | 4 | 2 | 3 | 4 |
| 3 | 2 | 5 | 2 | 4 | 5 | 4 | 5 | 5 |
| 1 | 2 | 2 | 2 | 5 | 5 | 5 | 4 | 5 |
| 3 | 4 | 4 | 0 | 4 | 4 | 2 | 4 | 5 |
| 5 | 3 | 4 | 4 | 3 | 4 | 5 | 2 | 4 |
| 4 | 2 | 3 | 4 | 1 | 9 | 0 | 3 | 3 |
| 0 | 5 | 2 | 5 | 2 | 5 | 5 | 5 | 3 |
| 0 | 4 | 5 | 1 | 5 | 5 | 5 | 3 | 2 |
| 3 | 1 | 2 | 2 | 1 | 5 | 5 | 5 | 4 |
| 2 | 1 | 4 | 3 | 5 | 5 | 0 | 3 | 5 |
| 2 | 5 | 5 | 1 | 5 | 4 | 3 | 1 | 1 |
| 0 | 1 | 3 | 0 | 5 | 2 | 4 | 5 | 5 |
| 3 | 5 | 5 | 2 | 2 | 5 | 5 | 0 | 5 |
| 2 | 1 | 2 | 2 | 4 | 3 | 3 | 4 | 5 |
| 2 | 4 | 5 | 1 | 2 | 5 | 5 | 1 | 3 |
| 3 | 2 | 4 | 5 | 2 | 3 | 0 | 1 | 4 |
| 1 | 4 | 5 | 4 | 3 | 5 | 2 | 5 | 1 |
| 4 | 1 | 2 | 4 | 5 | 4 | 2 | 5 | 4 |
| 0 | 4 | 5 | 5 | 1 | 5 | 5 | 4 | 5 |
| 4 | 3 | 3 | 2 | 0 | 3 | 0 | 3 | 3 |
| 3 | 0 | 2 | 1 | 5 | 4 | 5 | 5 | 3 |
| 4 | 5 | 4 | 5 | 3 | 3 | 1 | 0 | 2 |
| 5 | 4 | 4 | 3 | 2 | 3 | 4 | 4 | 5 |
| 2 | 5 | 4 | 5 | 5 | 5 | 4 | 5 | 2 |
| 0 | 5 | 0 | 4 | 5 | 3 | 4 | 4 | 4 |
| 5 | 2 | 3 | 5 | 5 | 5 | 4 | 1 | 5 |
| 4 | 5 | 1 | 2 | 2 | 3 | 3 | 4 | 2 |
| 5 | 3 | 3 | 5 | 4 | 4 | 5 | 5 | 5 |
| 4 | 5 | 2 | 0 | 1 | 4 | 2 | 3 | 4 |
| 3 | 5 | 5 | 2 | 4 | 5 | 5 | 2 | 5 |
| 1 | 4 | 2 | 2 | 3 | 4 | 5 | 4 | 5 |
| 3 | 4 | 4 | 0 | 4 | 4 | 2 | 4 | 5 |
| 0 | 4 | 4 | 4 | 5 | 2 | 5 | 2 | 0 |
| 4 | 3 | 1 | 5 | 1 | 3 | 5 | 3 | 3 |
| 0 | 5 | 4 | 1 | 2 | 5 | 5 | 0 | 5 |
| 0 | 5 | 5 | 1 | 5 | 5 | 0 | 3 | 2 |
| 3 | 1 | 2 | 2 | 1 | 5 | 4 | 5 | 4 |
| 2 | 1 | 4 | 3 | 5 | 5 | 3 | 3 | 5 |
| 2 | 5 | 5 | 1 | 5 | 3 | 5 | 1 | 4 |
| 5 | 1 | 3 | 0 | 2 | 4 | 1 | 3 | 5 |
| 3 | 4 | 5 | 2 | 4 | 5 | 5 | 0 | 5 |
| 2 | 1 | 2 | 2 | 4 | 3 | 5 | 4 | 5 |
| 2 | 5 | 5 | 5 | 2 | 2 | 1 | 1 | 3 |
| 3 | 4 | 4 | 3 | 2 | 2 | 0 | 1 | 4 |
| 1 | 4 | 5 | 4 | 5 | 5 | 3 | 5 | 1 |
| 4 | 1 | 2 | 4 | 1 | 2 | 4 | 5 | 5 |
| 0 | 1 | 5 | 5 | 1 | 5 | 4 | 4 | 5 |
| 4 | 4 | 3 | 2 | 0 | 3 | 5 | 3 | 5 |
| 3 | 0 | 2 | 5 | 1 | 4 | 2 | 5 | 4 |
| 4 | 5 | 0 | 2 | 3 | 3 | 5 | 0 | 3 |
| 5 | 4 | 4 | 3 | 5 | 5 | 2 | 4 | 1 |
| 2 | 5 | 4 | 5 | 5 | 4 | 4 | 5 | 2 |
| 0 | 4 | 5 | 4 | 5 | 4 | 4 | 4 | 4 |
| 5 | 2 | 3 | 5 | 5 | 5 | 5 | 1 | 5 |
| 0 | 5 | 5 | 2 | 2 | 3 | 3 | 4 | 2 |
| 5 | 3 | 3 | 5 | 4 | 5 | 5 | 5 | 5 |
| 4 | 2 | 2 | 5 | 5 | 5 | 2 | 3 | 5 |
| 3 | 3 | 5 | 5 | 4 | 5 | 1 | 2 | 4 |
| 1 | 2 | 2 | 2 | 3 | 4 | 5 | 4 | 5 |
| 3 | 4 | 4 | 0 | 4 | 4 | 2 | 4 | 3 |
| 5 | 5 | 4 | 4 | 3 | 3 | 5 | 2 | 4 |
| 4 | 2 | 1 | 4 | 1 | 4 | 5 | 3 | 3 |
| 0 | 5 | 2 | 1 | 5 | 5 | 4 | 0 | 0 |
| 0 | 4 | 5 | 1 | 5 | 3 | 0 | 3 | 5 |
| 3 | 4 | 2 | 2 | 1 | 5 | 3 | 5 | 4 |
| 2 | 4 | 4 | 3 | 5 | 5 | 5 | 3 | 5 |
| 2 | 5 | 5 | 5 | 5 | 3 | 3 | 1 | 1 |
| 0 | 5 | 3 | 0 | 2 | 4 | 5 | 3 | 5 |
| 3 | 5 | 5 | 2 | 4 | 5 | 2 | 0 | 0 |
| 2 | 5 | 2 | 2 | 4 | 3 | 3 | 4 | 5 |
| 2 | 5 | 5 | 1 | 2 | 4 | 5 | 1 | 3 |
| 3 | 2 | 4 | 3 | 5 | 3 | 4 | 4 | 4 |
| 5 | 4 | 5 | 4 | . | 5 | 5 | 5 | 5 |
| 4 | 4 | 2 | 4 | 1 | 3 | 5 | 5 | 4 |
| 0 | 1 | 5 | 5 | 1 | 4 | 4 | 4 | 5 |
| 4 | 4 | 3 | 5 | 0 | 3 | 0 | 3 | 4 |
| 3 | 5 | 2 | 1 | 1 | 4 | 2 | 5 | 0 |
| 4 | 5 | 5 | 2 | 5 | 3 | 5 | 0 | 2 |
| 5 | 4 | 5 | 3 | . | 3 | 2 | 4 | 1 |
| 2 | 5 | 5 | 5 | 5 | 5 | 3 | 5 | 2 |
| 0 | 3 | 5 | 4 | 5 | 4 | 5 | 4 | 4 |
| 1 | 2 | 3 | 5 | 5 | 5 | 3 | 1 | 5 |
| 0 | 5 | 1 | 2 | 2 | 4 | 5 | 4 | 2 |
| 0 | 3 | 3 | 5 | 4 | 5 | 4 | 5 | 2 |
| 1 | 5 | 2 | 0 | 1 | 4 | 2 | 3 | 4 |
| 3 | 4 | 5 | 2 | 4 | 5 | 1 | 2 | 0 |
| 1 | 2 | 5 | 2 | 5 | 1 | 5 | 4 | 5 |
| 3 | 4 | 4 | 0 | 4 | 4 | 5 | 4 | 3 |
| 5 | 4 | 4 | 4 | 3 | 5 | 5 | 2 | 0 |
| 4 | 2 | 1 | 4 | 1 | 4 | 4 | 3 | 3 |
| 0 | 5 | 2 | 5 | 2 | 5 | 5 | 0 | 5 |
| 0 | 4 | 5 | 1 | 5 | 3 | 5 | 3 | 2 |
| 3 | 4 | 2 | 2 | 1 | 5 | 3 | 5 | 4 |
| 2 | 1 | 4 | 3 | 5 | 5 | 5 | 3 | 5 |
| 2 | 5 | 5 | 1 | 5 | 5 | 3 | 1 | 1 |
| 0 | 1 | 3 | 0 | 5 | 4 | 1 | 3 | 5 |
| 3 | 4 | 5 | 2 | 4 | 5 | 4 | 0 | 4 |
| 2 | 1 | 5 | 2 | 4 | 3 | 3 | 4 | 5 |
| 2 | 4 | 5 | 1 | 2 | 2 | 1 | 1 | 3 |
| 3 | 2 | 4 | 5 | 2 | 2 | 5 | 1 | 4 |
| 0 | 4 | 5 | 4 | 4 | 2 | 2 | 5 | 1 |
| 4 | 5 | 4 | 4 | 1 | 2 | 5 | 5 | 5 |
| 0 | 5 | 5 | 5 | 1 | 4 | 4 | 4 | 5 |
| 4 | 4 | 3 | 2 | 0 | 3 | 5 | 3 | 4 |
| 3 | 5 | 2 | 1 | 5 | 4 | 2 | 5 | 5 |
| 4 | 5 | 5 | 2 | 3 | 3 | 1 | 5 | 2 |
| 5 | 4 | 5 | 3 | 0 | 5 | 2 | 4 | 1 |
| 2 | 5 | 5 | 5 | 5 | 4 | 0 | 5 | 2 |
| 0 | 3 | 0 | 5 | 5 | 5 | 4 | 4 | 4 |
| 0 | 2 | 3 | 4 | 5 | 5 | 0 | 1 | 5 |
| 0 | 5 | 3 | 2 | 5 | 3 | 4 | 4 | 2 |
| 5 | 5 | 3 | 4 | 4 | 4 | 3 | 5 | 4 |
| 0 | 3 | 2 | 0 | 1 | 4 | 5 | 3 | 4 |
| 3 | 2 | 5 | 2 | 4 | 5 | 3 | 2 | 4 |
| 1 | 2 | 2 | 5 | 3 | 3 | 5 | 4 | 5 |
| 3 | 5 | 4 | 4 | 4 | 4 | 5 | 4 | 5 |
| 0 | 5 | 4 | 4 | 5 | 2 | 5 | 2 | 0 |
| 4 | 2 | 1 | 4 | 4 | 3 | 0 | 3 | 3 |
| 0 | 5 | 2 | 4 | 2 | 5 | 5 | 5 | 4 |
| 0 | 5 | 5 | 1 | 5 | 4 | 4 | 3 | 2 |
| 3 | 3 | 2 | 2 | 1 | 5 | 3 | 5 | 4 |
| 2 | 4 | 4 | 4 | 5 | 5 | 5 | 3 | 5 |
| 2 | 5 | 5 | 1 | 5 | 5 | 3 | 4 | 4 |
| 0 | 4 | 3 | 4 | 2 | 4 | 5 | 3 | 5 |
| 3 | 5 | 5 | 2 | 0 | 5 | 2 | 0 | 4 |
| 2 | 3 | 3 | 4 | 5 | 3 | 3 | 5 | 5 |
| 2 | 5 | 5 | 5 | 2 | 3 | 1 | 1 | 3 |
| 3 | 2 | 4 | 3 | 2 | 2 | 5 | 1 | 4 |
| 5 | 4 | 5 | 4 | 4 | 2 | 4 | 5 | 4 |
| 4 | 5 | 2 | 4 | 1 | 3 | 4 | 5 | 5 |
| 0 | 4 | 5 | 5 | 1 | 4 | 5 | 4 | 5 |
| 4 | 4 | 3 | 2 | 5 | 3 | 4 | 3 | 4 |
| 3 | 4 | 2 | 1 | 4 | 4 | 5 | 5 | 4 |
| 0 | 5 | 4 | 2 | 3 | 3 | 3 | 0 | 2 |
| 0 | 5 | 4 | 3 | 0 | 3 | 2 | 4 | 4 |
| 2 | 5 | 4 | 5 | 5 | 3 | 3 | 5 | 4 |
| 0 | 3 | 0 | 4 | 5 | 4 | 4 | 4 | 4 |
| 5 | 2 | 3 | 5 | 5 | 5 | 4 | 5 | 5 |
| 4 | 5 | 1 | 2 | 2 | 3 | 5 | 4 | 2 |
| 0 | 3 | 3 | 4 | 4 | 5 | 5 | 5 | 3 |
| 4 | 2 | 2 | 4 | 5 | 5 | 2 | 3 | 4 |
| 3 | 5 | 5 | 4 | 4 | 5 | 1 | 2 | 4 |
| 1 | 2 | 2 | 2 | 3 | 5 | 5 | 4 | 5 |
| 3 | 5 | 4 | 0 | 4 | 4 | 2 | 4 | 4 |
| 5 | 5 | 4 | 4 | 3 | 2 | 5 | 2 | 5 |
| 4 | 2 | 1 | 5 | 4 | 4 | 0 | 3 | 3 |
| 0 | 5 | 2 | 4 | 2 | 5 | 5 | 0 | 0 |
| 0 | 4 | 5 | 1 | 5 | 3 | 5 | 3 | 5 |
| 3 | 5 | 2 | 2 | 1 | 5 | 3 | 5 | 4 |
| 2 | 4 | 4 | 4 | 5 | 5 | 4 | 3 | 5 |
| 2 | 5 | 5 | 1 | 5 | 3 | 3 | 5 | 5 |
| 5 | 1 | 3 | 4 | 2 | 4 | 5 | 3 | 5 |
| 1 | 4 | 5 | 2 | 5 | 5 | 4 | 4 | 5 |
| 2 | 4 | 2 | 2 | 4 | 3 | 3 | 4 | 5 |
| 2 | 4 | 5 | 1 | 2 | 2 | 4 | 1 | 4 |
| 3 | 5 | 4 | 3 | 4 | 2 | 4 | 1 | 5 |
| 1 | 4 | 5 | 4 | 4 | 5 | 5 | 5 | 1 |
| 4 | 5 | 2 | 5 | 1 | 2 | 2 | 5 | 0 |
| 0 | 4 | 5 | 5 | 1 | 5 | 4 | 4 | 5 |
| 4 | 4 | 3 | 4 | 0 | 3 | 5 | 3 | 4 |
| 3 | 0 | 2 | 4 | 1 | 4 | 5 | 5 | 4 |
| 4 | 5 | 0 | 2 | 5 | 3 | 3 | 0 | 2 |
| 5 | 4 | 4 | 3 | 0 | 3 | 2 | 4 | 1 |
| 2 | 5 | 4 | 5 | 5 | 5 | 4 | 5 | 4 |
| 0 | 5 | 0 | 4 | 5 | 4 | 5 | 4 | 4 |
| 5 | 5 | 3 | 5 | 5 | 5 | 5 | 1 | 5 |
| 4 | 5 | 1 | 5 | 4 | 3 | 3 | 4 | 2 |
| 5 | 3 | 3 | 5 | 5 | 5 | 2 | 5 | 4 |
| 4 | 3 | 2 | 0 | 1 | 4 | 2 | 3 | 5 |
| 3 | 2 | 5 | 2 | 4 | 5 | 1 | 2 | 5 |
| 1 | 4 | 5 | 4 | 3 | 5 | 5 | 4 | 5 |
| 3 | 0 | 5 | 4 | 4 | 4 | 2 | 4 | 3 |
| 5 | 4 | 4 | 4 | 3 | 2 | 5 | 2 | 4 |
| 4 | 5 | 1 | 4 | 5 | 4 | 5 | 3 | 3 |
| 0 | 5 | 2 | 4 | 2 | 5 | 5 | 0 | 5 |
| 0 | 3 | 5 | 1 | 5 | 1 | 4 | 3 | 5 |
| 3 | 5 | 2 | 5 | 1 | 5 | 3 | 5 | 4 |
| 2 | 4 | 4 | 3 | 5 | 5 | 3 | 3 | 5 |
| 2 | 5 | 5 | 4 | 5 | 5 | 3 | 1 | 4 |
| 5 | 3 | 4 | 0 | 5 | 3 | 1 | 3 | 5 |
| 3 | 4 | 5 | 2 | 0 | 5 | 2 | 0 | 4 |
| 2 | 1 | 2 | 2 | 4 | 3 | 5 | 4 | 5 |
| 2 | 3 | 5 | 1 | 2 | 5 | 5 | 1 | 3 |
| 3 | 5 | 4 | 3 | 2 | 5 | 0 | 5 | 4 |
| 5 | 4 | 5 | 4 | 0 | 4 | 2 | 5 | 5 |
| 4 | 3 | 2 | 4 | 5 | 2 | 5 | 5 | 0 |
| 0 | 4 | 5 | 5 | 1 | 3 | 4 | 4 | 5 |
| 4 | 3 | 3 | 2 | 0 | 3 | 5 | 3 | 5 |
| 2 | 5 | 4 | 5 | 1 | 4 | 2 | 5 | 4 |
| 4 | 5 | 3 | 2 | 3 | 3 | 5 | 0 | 2 |
| 1 | 4 | 4 | 3 | 0 | 3 | 2 | 4 | 5 |
| 2 | 5 | 4 | 5 | 5 | 5 | 5 | 5 | 2 |
| 0 | 3 | 5 | 4 | 5 | 5 | 1 | 4 | 5 |
| 5 | 2 | 5 | 5 | 5 | 5 | 5 | 1 | 5 |
| 4 | 5 | 5 | 5 | 2 | 3 | 5 | 4 | 5 |
| 1 | 3 | 5 | 5 | 4 | 4 | 2 | 5 | 5 |
| 4 | 4 | 2 | 0 | 1 | 4 | 2 | 5 | 5 |
| 2 | 4 | 5 | 2 | 5 | 5 | 1 | 2 | 5 |
| 1 | 2 | 2 | 2 | 3 | 4 | 5 | 4 | 5 |
| 3 | 4 | 4 | 5 | 4 | 4 | 2 | 4 | 3 |
| 0 | 5 | 4 | 4 | 3 | 4 | 5 | 2 | 0 |
| 4 | 2 | 4 | 4 | 1 | 3 | 4 | 3 | 5 |
| 0 | 5 | 4 | 1 | 2 | 5 | 5 | 5 | 0 |
| 0 | 4 | 5 | 1 | 5 | 3 | 0 | 3 | 2 |
| 3 | 3 | 2 | 2 | 1 | 5 | 3 | 5 | 4 |
| 2 | 4 | 4 | 3 | 5 | 5 | 5 | 3 | 5 |
| 2 | 5 | 5 | 1 | 5 | 3 | 5 | 1 | 5 |
| 1 | 5 | 3 | 4 | 2 | 5 | 1 | 5 | 5 |
| 2 | 4 | 5 | 2 | 0 | 5 | 5 | 5 | 5 |
| 2 | 5 | 2 | 2 | 4 | 3 | 3 | 4 | 5 |
| 2 | 3 | 5 | 1 | 2 | 4 | 1 | 1 | 3 |
| 3 | 3 | 4 | 3 | 2 | 5 | 5 | 1 | 5 |
| 1 | 4 | 5 | 4 | 5 | 2 | 2 | 5 | 1 |
| 0 | 5 | 2 | 4 | 1 | 5 | 4 | 5 | 5 |
| 0 | 5 | 5 | 5 | 1 | 4 | 4 | 4 | 5 |
| 2 | 4 | 3 | 2 | 0 | 4 | 0 | 3 | 4 |
